# Biological effect of tissue plasminogen activator (t-PA) and DNase intrapleural delivery in pleural infection patients

**DOI:** 10.1136/bmjresp-2019-000440

**Published:** 2019-09-23

**Authors:** Nikolaos I Kanellakis, John M Wrightson, Rob Hallifax, Eihab O Bedawi, Rachel Mercer, Maged Hassan, Rachelle Asciak, Emma Hedley, Melissa Dobson, Tao Dong, Ioannis Psallidas, Najib M Rahman

**Affiliations:** 1 Oxford Centre for Respiratory Medicine, Churchill Hospital, Oxford University Hospitals NHS Foundation Trust, Oxford, Oxfordshire, UK; 2 Laboratory of Pleural and Lung Cancer Translational Research, Nuffield Department of Medicine, University of Oxford, Oxford, Oxfordshire, UK; 3 National Institute for Health Research Oxford Biomedical Research Centre, University of Oxford, Oxford, Oxfordshire, UK; 4 Oxford Respiratory Trials Unit, Nuffield Department of Medicine, University of Oxford, Oxford, Oxfordshire, UK; 5 Centre for Translational Immunology, Chinese Academy of Medical Sciences Oxford Institute, Nuffield Department of Medicine, University of Oxford, Oxford, UK; 6 MRC Human Immunology Unit, Weatherall Institute of Molecular Medicine, University of Oxford, Oxford, UK

**Keywords:** pleural infection, empyema, t-PA, fibrinolytics, DNase

## Abstract

**Background:**

Pleural infection (PI) is a major global disease with an increasing incidence, and pleural fluid (PF) drainage is essential for the successful treatment. The MIST2 study demonstrated that intrapleural administration of tissue plasminogen activator (t-PA) and DNase, or t-PA alone increased the volume of drained PF. Mouse model studies have suggested that the volume increase is due to the interaction of the pleura with the t-PA via the monocyte chemoattractant protein 1 (MCP-1) pathway. We designed a study to determine the time frame of drained PF volume induction on intrapleural delivery of t-PA±DNase in humans, and to test the hypothesis that the induction is mediated by the MCP-1 pathway.

**Methods:**

Data and samples from the MIST2 study were used (210 PI patients randomised to receive for 3 days either: t-PA and DNase, t-PA and placebo, DNase and placebo or double placebo). PF MCP-1 levels were measured by ELISA. One-way and two-way analysis of variance (ANOVA) with Tukey’s post hoc tests were used to estimate statistical significance. Pearson’s correlation coefficient was used to assess linear correlation.

**Results:**

Intrapleural administration of t-PA±DNase stimulated a statistically significant rise in the volume of drained PF during the treatment period (days 1–3). No significant difference was detected between any groups during the post-treatment period (days 5–7). Intrapleural administration of t-PA increased MCP-1 PF levels during treatment; however, no statistically significant difference was detected between patients who received t-PA and those who did not. PF MCP-1 expression was not correlated to the drug given nor the volume of drained PF.

**Conclusions:**

We conclude that the PF volume drainage increment seen with the administration of t-PA does not appear to act solely via activation of the MCP-1 pathway.

**Key messages**

- The aim of this study was to assess the hypothesis that induction of pleural fluid is mediated via the monocyte chemoattractant protein 1 (MCP-1) pathway.
- The pleural fluid volume drainage increment seen with the administration of tissue plasminogen activator does not appear to act solely via activation of the MCP-1 pathway.
- This is the first study to use human samples to test the MCP-1 fluid production hypothesis, and suggests the presence of additional pathways.

## Introduction

Pleural infection (PI; empyema or complicated parapneumonic effusion) is a major and complex global disease. PI is an age-old clinical problem, which was accurately described by Hippocrates (460–370 BC) and has been responsible for numerous deaths ever since. Every year PI affects 80 000 patients in the UK and the USA and there is evidence suggesting that the incidence of the disease is increasing in both adult and paediatric populations.^1–4^ Standard medical treatment of PI relies on appropriate antibiotic administration, drainage of the infected pleural fluid (PF) and nutritional support.^5^

Despite diagnostic and therapeutic advances over recent years, PI remains a severe illness associated with poor clinical outcome and high morbidity and mortality. Approximately 20% of PI patients require surgery as a rescue treatment when antibiotic administration and PF drainage through a chest tube fail and the disease progresses.^5 6^ The 1-year mortality rate for the general population of PI patients is around 20%, whereas for aged and frail patients, it is above 30%.^7 8^ Hospitalisation rates have doubled and the median length of hospitalisation ranges between 12 and 15 days, with 25% of patients remaining in hospital for more than 1 month.^8–11^ As a result, PI is associated with a heavy socioeconomic burden with the related healthcare cost estimated at US$5000 per patient.^12^

As the disease progresses, intrapleural levels of fibrinolysis inhibitors, including tissue plasminogen activator inhibitor 1 (PAI-1), rise leading to the arrest of fibrinolytic activity and consequently to the intrapleural accumulation of fibrin.^13 14^ The development of pleural fibrinous septations partitions the pleural space into several locules that prevent drainage of PF and thus increase the risk of requirement for surgery as rescue treatment. The intrapleural administration of fibrinolytics has been advocated as a treatment that cleaves fibrin, and therefore, improves chest-tube drainage and reduces the need for surgery. Notably, human and animal data suggest that intrapleural delivery of tissue plasminogen activator (t-PA) stimulates an increase in PF output.^15 16^

The MIST2 randomised clinical trial (RCT) tested the effect of intrapleural administration of t-PA to cleave septations, and DNase to reduce viscosity of PF due to extracellular DNA.^17^ The results demonstrated that intrapleural delivery of t-PA and DNase, or t-PA alone increased the volume of drained PF, and t-PA DNase was associated with reduced length of hospital stay and need for surgical referral at 3 months.^18^ However, the time frame of the intrapleural drug activity remains unknown, and identification of the intrapleural t-PA±DNase activity period is essential to improve patient management. Moreover, the molecular pathways by which fibrinolytics promote increased PF output and facilitate the drainage of pleural space are unclear.

Monocyte chemoattractant protein 1 (MCP-1), also known as chemokine ligand 2 (CCL-2), is a chemokine that has been associated with inflammation, endothelial permeability, malignant pleural effusion and pleural injury repair mechanisms.^19–21^ A study, based on mouse in vivo and human in vitro data, suggested that PF volume induction is due to the interaction of the pleura with t-PA via the MCP-1 pathway.^22^ Lansley *et al* reported that the inhibition of MCP-1 activity decreased the volume of PF induction on intrapleural delivery of t-PA. Data from a clinical trial suggested a benefit for PI patients that received pleural saline irrigation.^23^ These two studies in combination suggest that the stimulation of pleural effusion formation (‘pleural weep’) caused by intrapleural t-PA delivery may trigger a therapeutic lavage that enables clearance of the pleural cavity. However, the MCP-1 hypothesis has not been confirmed in human PI patients.

We designed and conducted a study with the aim to identify the time frame of intrapleural t-PA±DNase activity and to assess the hypothesis that MCP-1 pathway mediates the drained PF volume induction in human PI patients following intrapleural delivery of t-PA.

## Methods

### Samples

For the present study, we used the PF samples of the MIST2 RCT.^18^ PF samples were taken at randomisation and daily thereafter (where available), processed with sodium citrate and stored in −80°C freezers.

### PF drainage records

For the purposes of the MIST2 study, PF drainage was recorded daily and used to determine the activity period of intrapleural t-PA±DNase.

### Patients

Two hundred and twenty PI patients provided written consent to participate into the MIST2 study and were randomly assigned to receive intrapleurally for 3 days either: t-PA and DNase, t-PA and placebo, DNase and placebo or double placebo. The daily doses of t-PA and DNase were 10 and 5 mg, respectively. Full details of the study are available in the original publication.

### PF MCP-1 protein measurement

PF MCP-1 levels were measured by ELISA (Human CCL2/MCP-1 Quantikine ELISA Kit, DCP00, R&D Systems). All available samples from different groups (t-PA and DNase, t-PA and placebo, DNase and placebo, double placebo) and different time points (randomisation, treatment, post-treatment) were included in the analysis. Triplicates were used for protein measurement of each PF sample and the samples of the standard curve. Only samples, which had not been previously thawed, were used for protein measurements.

### Patient and public involvement

Patients and public were involved to assess the aims of the study. Patients and public were not involved for recruitment and conduct of the study. A lay summary of the results will be shared with the public.

### Statistics

All analyses were performed using GraphPad Prism (V.7.0; GraphPad Software, La Jolla, California, USA). Data are presented as mean±SD or Box-and-whisker plots (Tukey boxes) as described. One-way and two-way ANOVA with Tukey’s post-tests were used to estimate the statistical significance. Pearson’s correlation coefficient was used to estimate the linear correlation. Statistical significance was taken at the 5% level.

## Results

### The volume of drained PF is significantly increased during intrapleural administration of t-PA and DNase or t-PA and placebo

**Figure 1:**
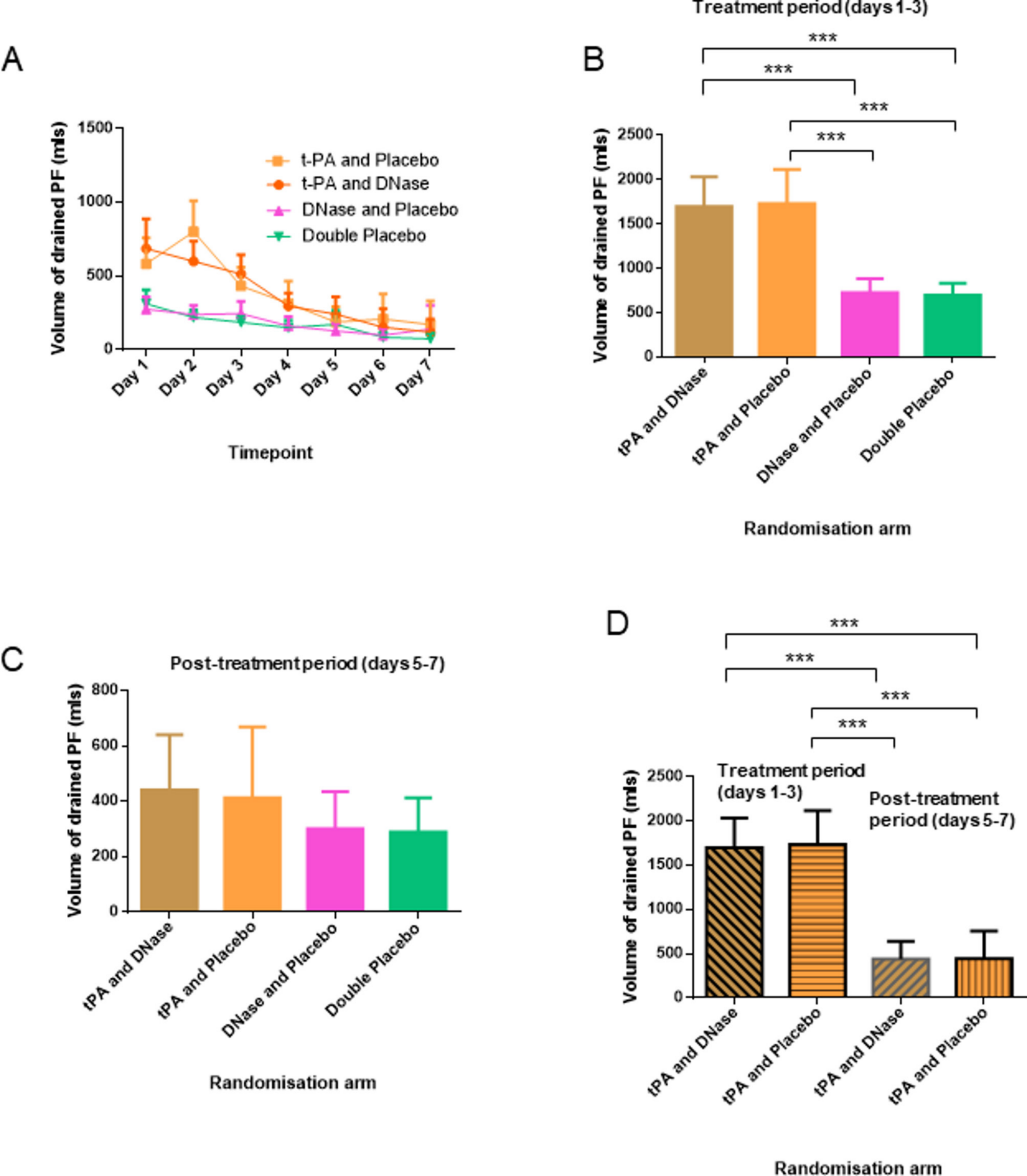
Intrapleural administration of t-PA and DNase increases the volume of output pleural fluid (PF). (A) Daily output of PF volume in mL for each randomisation arm of the MIST2 study: t-PA and DNase, t-PA and placebo, DNase and placebo and double placebo. during treatment period (days 1–3) groups receiving t-PA±DNase demonstrated increased volume of drained pleural fluid compared with non-t-PA receiving groups for the same period and all groups for post-treatment period (days 5–7). Data are presented as mean±95% CI. (B) Output of PF volume in mL for each randomisation arm during treatment period (days 1–3). The groups that received t-PA alone or t-PA and DNase exhibited increased volume of drained PF. Data are presented as mean±95% CI. ***Denotes p<0.001 for the indicated comparisons by one-way ANOVA with Tukey’s post-tests. (C) Output of PF volume in mL for each randomisation arm during post-treatment period (days 5–7). No statistical significant differences were found between groups by one-way ANOVA with Tukey’s post-tests. Data are presented as mean±95% CI. (D) Output of PF volume in mL for the groups that received t-PA and DNase and t-PA and placebo during the treatment and post-treatment periods. While treatment was ongoing, patients receiving intrapleural t-PA exhibited higher volume of drained PF compared with the post-treatment period. Data are presented as mean±95% CI. ***Denotes p<0.001 for the indicated comparisons by one-way ANOVA with Tukey’s post hoc tests. ANOVA, analysis of variance; t-PA, tissue plasminogen activator.

The MIST2 RCT demonstrated that intrapleural administration of t-PA in PI patients improved PF drainage volume.^18^ We analysed the daily volume of PF output to identify the time frame of the volume increase (figure 1A). We discovered that during the treatment period (days 1–3) patients who received intrapleural t-PA and DNase (mean: 1700 mL, 95% CI 1356.0 to 2000.0) or t-PA and placebo (mean: 1733 mL, 95% CI 1349.0 to 2117.0) exhibited statistically significant increased (ANOVA p<0.001) volume of drained PF compared with patients managed with DNase and placebo (mean: 725 mL, 95% CI 566.0 to 884.0) or double placebo (mean: 699 mL, 95% CI 562.3 to 836.8) (figure 1A, B). However, there were no statistically significant differences of the drained PF volume (ANOVA p>0.05) between any of the groups during the post-treatment period (days 5–7) (figure 1A, C).

Subsequently, we compared the volume of drained PF between treatment and post-treatment period for each randomisation arm. PI patients randomised to receive daily doses of t-PA and DNase or t-PA and placebo exhibited statistically significant larger (ANOVA p<0.001) volumes of PF output during treatment compared with the post-treatment period (figure 1D). These data combined suggest that administration of t-PA, with or without DNase, does not have an effect on the volume of drained PF during the post-treatment period and only occurs during (within 12 hours of) administration of t-PA±DNase.

### PF MCP-1 levels are not correlated to the drug given nor the volume of drained PF

The exact molecular mechanism by which intrapleural delivery of t-PA increases the output volume of PF remains unknown. Lansley *et al* delivered fibrinolytics to the pleural space of mice and proposed that t-PA induces the intrapleural levels of MCP-1 and reported a correlation between the MCP-1 levels and the volume of pleural effusion.^22^ MCP-1 has been identified as a promoter of vascular permeability and an inflammatory cell recruitment factor in malignant pleural effusion.^21^ To this end, the authors suggested that these abilities may explain the PF formation.

**Figure 2:**
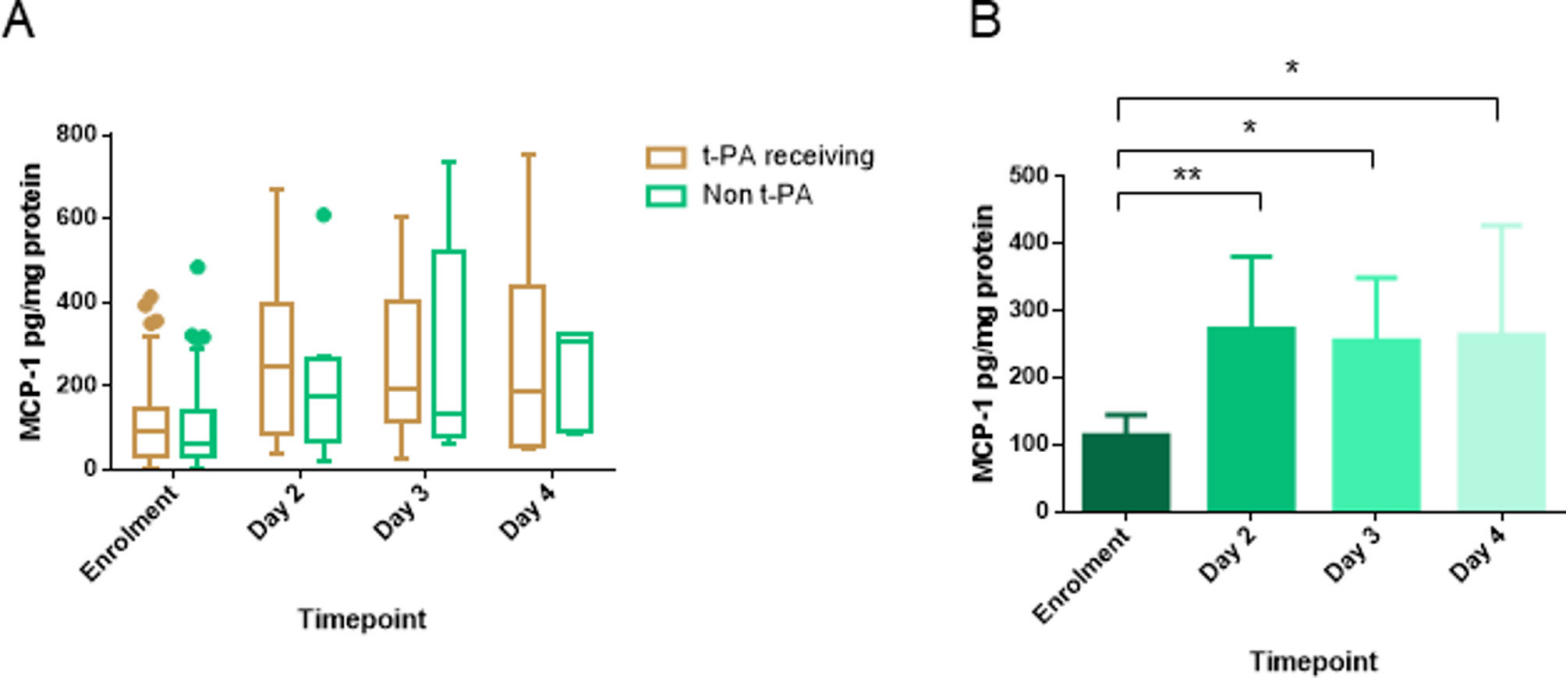
The intrapleural delivery of t-PA and DNase was not found correlated to MCP-1 PF levels. (A) MCP-1 PF levels comparing the t-PA (t-PA alone, t-PA and DNase) versus the non-t-PA (DNase, placebo) receiving groups. Data are presented as Tukey’s boxes. No statistical significant differences were detected with two-way ANOVA with multiple comparisons between the t-PA and non-t-PA receiving groups. (B) MCP-1 PF levels increased significantly in patients that received t-PA±DNase during days 2–4 compared with enrolment. Data are presented as mean±95% CI. **Denotes p<0.01 and *p<0.05 for the indicated comparisons by one-way ANOVA with Tukey’s post-tests. ANOVA, analysis of variance; MCP-1, monocyte chemoattractant protein 1; PF, pleural fluid; t-PA, tissue plasminogen activator.

To assess the potential role of MCP-1 in PF formation on intrapleural treatment with t-PA, we measured MCP-1 protein levels on MIST2 study PF samples and assessed the correlation between MCP-1 protein expression and the volume of PF output. No increase in MCP-1 expression (two-way ANOVA p>0.05) was detected between PI patients who received t-PA and DNase or t-PA and placebo compared with patients from the non t-PA treatment groups (figure 2A). Interestingly, when we analysed only the group of patients who received t-PA±DNase a statistically significant increase of MCP-1 levels (one-way ANOVA p<0.05) was detected (figure 2B). However, this increase was not sufficient to demonstrate statistical significance between the t-PA and non t-PA receiving groups during the period of treatment. MCP-1 PF levels were not correlated to PF output volume on intrapleural administration of t-PA (Pearson correlation coefficient, R square=0.006, p=0.39). These data suggest that MCP-1 is likely to be associated with the formation process of the excess PF, but is unlikely to be the sole mechanism of action.

## Discussion

PI is a severe disease, with an increasing incidence and high mortality that often requires invasive treatments.^6 18^ The accumulation of fibrin and extracellular DNA disturb easy drainage of the infected PF. The MIST2 study demonstrated that intrapleural administration of t-PA and DNase in PI patients improved PF drainage and reduced surgical referral rate. A mouse PI model suggested that the intrapleural delivery of t-PA induces the levels of MCP-1, which subsequently promotes the formation of PF. We designed and conducted the first study to assess the MCP-1 hypothesis in humans. We measured the MCP-1 PF levels of MIST2 samples and examined the probable correlation between protein levels and volume of the PF output. Our data suggest that MCP-1 is likely to be associated with the formation process of excess PF, but is unlikely to be the sole mechanism of action.

The comparison of the volume of drained PF between the periods of treatment and post-treatment revealed that intrapleural delivery of t-PA±DNase increases PF volume drained solely during the treatment period. During the post-treatment period, no statistical differences in PF volume were observed between groups. The effect of t-PA±DNase was observed only during the period of drug delivery. Following t-PA±DNase treatment, the volume of PF output decreased and reached a level close to the non-t-PA treated patients (figure 1). Interestingly, whereas during the treatment period, there was a statistically significant increment in PF MCP-1 levels for patients who received t-PA±DNase compared with enrolment (baseline), no differences were detected when compared with the non t-PA (placebo±DNase) groups (figure 2). Moreover, protein levels of MCP-1 were not correlated to volume of drained PF.

This study has limitations regarding the stability of the proteins after a long-term freeze period. The number of freeze-thaw cycles affects stability of the MCP-1 recombinant protein.^24^ This may be an issue for native MCP-1 stability in our samples, thus, we used samples that have not been thawed in the past.

The trend in MCP-1 PF levels suggests that the MCP-1 pathway partially affects the induction of excess PF, however, it is likely that there are more key molecular pathways involved. A high-throughput protein profiling assay study is now needed to identify the molecular pathways that drive PF formation following intrapleural delivery of t-PA. The complexity of the human pleural space compared with the murine model may explain the differences between the data presented herein and the results from Lansley *et al*.^22^ Importantly, mouse and human PAI-1 and fibrin receptors have a diverse structure and as a consequence the mouse model of PI has limitations, hampering the translation of the mouse findings to human biology.^14^

## Data Availability

Data are available on reasonable request.
